# BCL2 and hsa-miR-181a-5p are potential biomarkers associated with papillary thyroid cancer based on bioinformatics analysis

**DOI:** 10.1186/s12957-019-1755-9

**Published:** 2019-12-16

**Authors:** Cong Zhang, Chunrui Bo, Lunhua Guo, Pingyang Yu, Susheng Miao, Xin Gu

**Affiliations:** 1 Department of Head and Neck Surgery, Harbin Medical University Cancer Hospital, Harbin, 150000 Heilongjiang People’s Republic of China; 2 Department of Neurology, the Second Affiliated Hospital, Harbin Medical University, Harbin, 150081 Heilongjiang China

**Keywords:** Papillary thyroid cancer (PTC), Differentially expressed genes (DEGs), Deregulated miRNAs, BCL2, hsa-miR-181a-5p

## Abstract

**Background:**

The morbidity of thyroid carcinoma has been rising worldwide and increasing faster than any other cancer type. The most common subtype with the best prognosis is papillary thyroid cancer (PTC); however, the exact molecular pathogenesis of PTC is still not completely understood.

**Methods:**

In the current study, 3 gene expression datasets (GSE3678, GSE3467, and GSE33630) and 2 miRNA expression datasets (GSE113629 and GSE73182) of PTC were selected from the Gene Expression Omnibus (GEO) database and were further used to identify differentially expressed genes (DEGs) and deregulated miRNAs between normal thyroid tissue samples and PTC samples. Then, Gene Ontology (GO) and pathway enrichment analyses were conducted, and a protein-protein interaction (PPI) network was constructed to explore the potential mechanism of PTC carcinogenesis. The hub gene detection was performed using the CentiScaPe v2.0 plugin, and significant modules were discovered using the MCODE plugin for Cytoscape. In addition, a miRNA-gene regulatory network in PTC was constructed using common deregulated miRNAs and DEGs.

**Results:**

A total of 263 common DEGs and 12 common deregulated miRNAs were identified. Then, 6 significant KEGG pathways (*P* < 0.05) and 82 significant GO terms were found to be enriched, indicating that PTC was closely related to amino acid metabolism, development, immune system, and endocrine system. In addition, by constructing a PPI network and miRNA-gene regulatory network, we found that hsa-miR-181a-5p regulated the most DEGs, while *BCL2* was targeted by the most miRNAs.

**Conclusions:**

The results of this study suggested that hsa-miR-181a-5p and *BCL2* and their regulatory networks may play important roles in the pathogenesis of PTC.

## Background

The morbidity of thyroid carcinoma has been rising worldwide and increasing faster than any other type of cancer, mainly due to the increasing use of diagnostic equipment [1]. It was reported that in 2017 thyroid cancer was the fifth most common cancer among American women [2]. Remarkably, thyroid cancer is 3 times more common in women than in men [2]. Similarly, thyroid cancer was the fastest growing cancer among Chinese from 1988 to 2013. Between 1988 and 2013, the incidence of thyroid cancer increased by an average of 14.73% per year in men and 18.98% per year in women [3]. Papillary thyroid cancer (PTC) is the most common subtype of differentiated thyroid cancers (DTCs), with the best overall prognosis [4]. However, the biggest challenge in treating PTC is to identify an easy method for the early recognition of benign or malignant nodules and the detection of overtreatment.

In recent years, a number of risk genes of PTC have been identified, including Cbp/p300 interacting transactivator with Glu/Asp rich carboxy-terminal domain 1 (*CITED1*) [5], LDL receptor related protein 4 (*LRP4*) [6] and tektin 4 (*TEKT4*) [7], whose downregulation can significantly inhibit the proliferation, migration, and invasion of PTC cells. Interleukin 17 receptor A (*IL17RA*) polymorphisms, which play an important role in tumor development, have been found to influence both the unilateral and bilateral development of PTC [8]. In addition, increased expression of flavin-containing monooxygenase 1 (encoded by the *FMO1* gene) could serve as a biomarker that independently predicts favorable recurrence-free survival in classical PTC patients [9]. These observations suggest that an increasing number of genes are crucial for the pathogenesis of PTC.

In addition, miRNAs also play an important role in the pathogenesis of various cancers, especially PTC. For example, miR-524-5p can inhibit cell migration, invasion, and apoptosis by targeting *FOXE1* and *ITGA3* in PTC [10]. In addition, miR-215 was found to target *ARFGEF1* and inhibit the proliferation and metastasis of PTC by regulating the epithelial-mesenchymal transformation [11]. Furthermore, miR-509 [12], miR-1270 [13], miR-128 ,[14] and many other miRNAs can inhibit PTC by targeting specific genes. These studies focused on one specific gene or miRNA; however, the comprehensive view of how these miRNAs and genes affect PTC remains unknown. The aim of our study was to screen significant gene and miRNA changes through bioinformatics methods to provide guidance for the study of PTC mechanisms and clinical treatment.

In this study, 3 gene expression datasets (GSE3678, GSE3467, and GSE33630) and 2 miRNA expression datasets (GSE113629 and GSE73182) (Sample analysis was shown in Additional file 1: Figure S1) of PTC were selected from the GEO database that were further used to identify DEGs and deregulated miRNAs between normal thyroid tissue samples and PTC samples. As a result, 263 DEGs and 12 deregulated miRNAs were identified based on the criteria we set. Then, GO and pathway enrichment analyses were conducted, and a PPI network was constructed to explore the potential mechanism of PTC carcinogenesis. The hub gene detection was performed using the CentiScaPe v2.0 plugin, and significant modules were discovered using the MCODE plugin for Cytoscape. In addition, a miRNA-gene regulatory network of PTC was constructed using common deregulated miRNAs and DEGs, and we found that hsa-miR-181a-5p regulated the most DEGs, while *BCL2* was targeted by the most miRNAs in this network. However, the specific mechanisms of how hsa-miR-181a-5p could regulate *BCL2* need further experiments. In conclusion, hsa-miR-181a-5p and *BCL2* are expected to be distinctive biomarkers of benign or malignant tumors and potential therapeutic targets of PTC.

## Methods

### Acquisition of gene and miRNA expression profile microarray data

The microarray data were acquired from the Gene Expression Omnibus (GEO) database (www.ncbi.nlm.nih.gov/geo) [15]. Three gene expression datasets (GSE3678, GSE3467, and GSE33630) and 2 miRNA expression datasets (GSE113629 and GSE73182) of PTC were included in this study.

Dataset GSE3678 included 7 PTC samples and 7 paired normal thyroid tissue samples; dataset GSE3467 included 9 PTC patients with paired tumor and normal thyroid tissue; and dataset GSE33630 included 49 PTC samples and 45 normal thyroid tissue samples. These 3 gene expression datasets were all based on the platform of GPL570 [HG-U133_Plus_2] Affymetrix Human Genome U133 Plus 2.0 Array [16–19].

The miRNA dataset GSE113629, based on the GPL24741 Agilent-070156 Human_miRNA_V21.0_Microarray 046064 platform, included matched neoplasms and normal thyroid tissues from 5 patients with PTC. The GSE73182 dataset based on the GPL20194 Agilent-035758 Human miRBASE 16.0 plus 031181 platform included 19 primary papillary thyroid carcinomas and 5 normal thyroids [20, 21].

### Identification of DEGs and deregulated miRNA

The interactive web tool GEO2R (www.ncbi.nlm.nih.gov/geo/geo2r) was used to screen the DEGs and deregulated miRNAs between normal thyroid tissue samples and PTC samples [15]. The Benjamin and Hochberg false discovery rate (FDR) method was used to correct the adjusted *P* value and correct the occurrence of false positive results. The cutoff standard was defined as *P* value< 0.01, adjusted *P* value < 0.01 and |logFC| > 1.

### GO terms and KEGG pathway enrichment analysis

Pathway data were obtained from the Kyoto Encyclopedia of Genes and Genomes (KEGG) database (https://www.kegg.jp/) [22] to examine specific pathways. To identify the Gene Ontology (GO) annotation and pathways in which DEGs were enriched, functional annotation tools were used for GO terms and KEGG pathway enrichment analysis in Database for Annotation, Visualization and Integrated Discovery (DAVID) (https://david.ncifcrf.gov/) [23]. The significance level of KEGG pathway enrichment was calculated using a cutoff of *P* value < 0.05. A GO term was considered significantly enriched if it showed a *P* value < 0.05.

### Construction of the PPI network

The PPI network of DEGs was identified using the Search Tool for the Retrieval of Interacting Genes (STRING) (http://string-db.org/) database [24]. Subsequently, Cytoscape software (v 3.6.1) was used to visualize the PPI network.

### Topological measurements of the PPI network

Several common topological measurements were investigated to reveal the basic features of the PPI network. The node degree, neighborhood connectivity, topological coefficients, and clustering coefficients were analyzed for the whole network.

### Hub gene identification and module analysis of the PPI network

The hub genes in this PPI network were defined as nodes with a connective degree > 10 and identified using the CentiScaPe v2.0 plugin for Cytoscape [25]. The most significant modules in the PPI network were identified by the Molecular Complex Detection (MCODE) plugin [26] with MCODE scores ≥ 5, degree cutoff = 2, node score cutoff = 0.2, Max depth = 100 and k-core = 3.

### Construction of a network of deregulated miRNAs targeting DEGs

The miRNA-gene target data were extracted from miRTarBase [27] (http://mirtarbase.mbc.nctu.edu.tw/php/index.php). When miRNA-gene pairs showed strong evidence of interaction in humans and matched the DEGs we identified, they were selected. Subsequently, Cytoscape software (v 3.6.1) was used to visualize the miRNA-gene network.

## Results

### Identification of DEGs and deregulated miRNAs in PTC

**Fig. 1:**
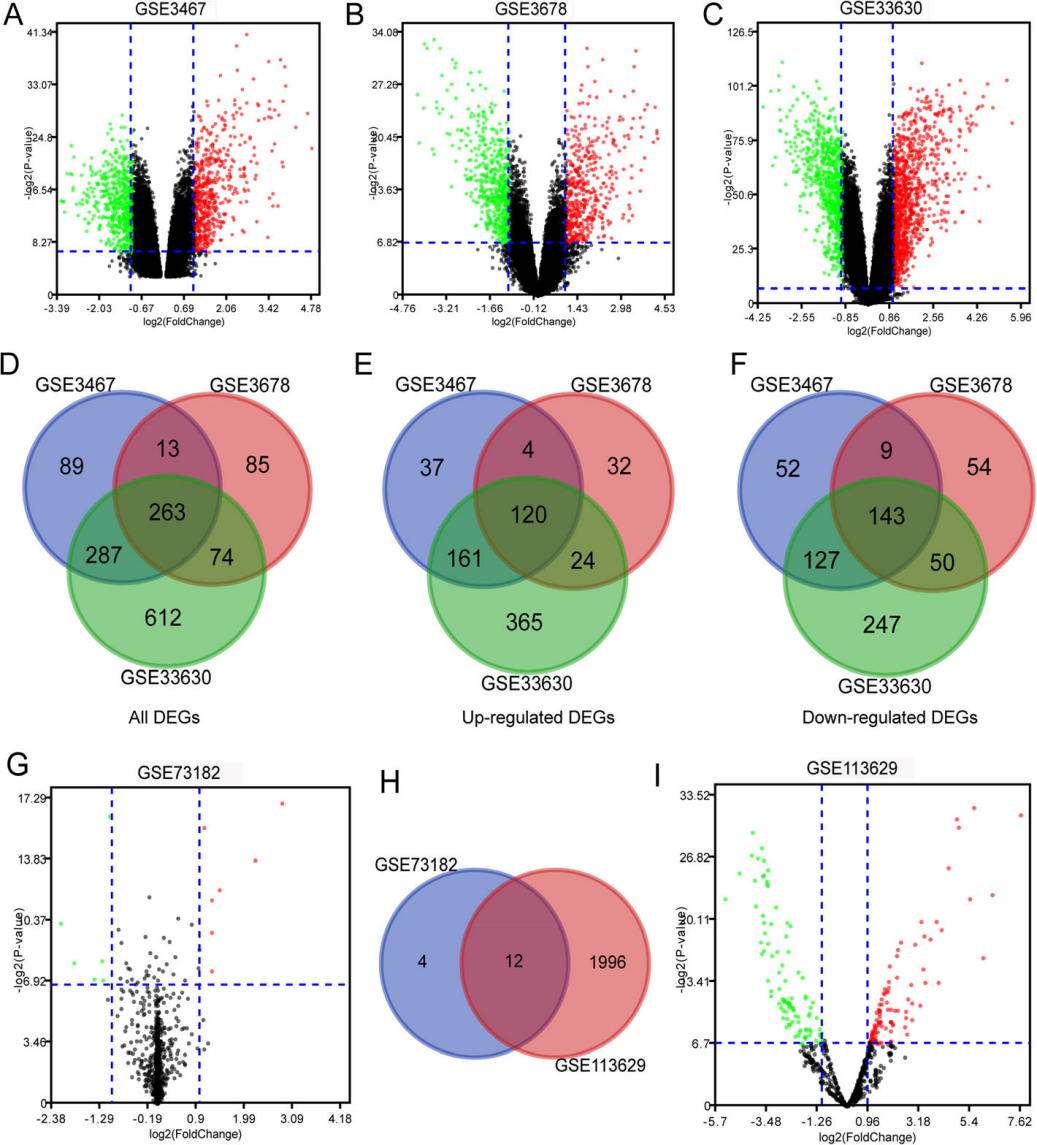
Volcano plot and Venn diagram of DEGs and deregulated miRNAs in gene/miRNA expression profiling datasets. **a**–**c** Volcano plots of DEGs in normal thyroid tissue and PTC samples in the GSE3467, GSE3678, and GSE33630 datasets. **d**–**f** Venn diagrams illustrating the number of all upregulated and downregulated DEGs in three gene expression datasets. The intersection in the center represents the common DEGs among the three datasets. **g**, **i** Volcano plots of deregulated miRNAs in normal thyroid tissue and PTC samples in GSE73182 and GSE113629 datasets, respectively. **h** Venn diagrams of all deregulated miRNAs in the two datasets. The intersection in the center represents the common deregulated miRNAs between the two datasets. DEGs and deregulated miRNAs were selected by *P* value < 0.01 and |logFC| > 1. The *x*-axis shows the fold-change in gene/miRNA expression, and the *y*-axis shows the statistical significance of the differences. Colors represent different genes/miRNAs: black for genes/miRNAs without significantly different expression, red for significantly upregulated genes/miRNAs and green for significantly downregulated genes/miRNAs. PTC, papillary thyroid cancer; FC, fold change

The gene expression datasets GSE3678, GSE3467, and GSE33630 were acquired from the GEO database. DEGs between normal thyroid tissue and PTC samples were screened using GEO2R. As a result, 436, 653, and 1237 DEGs were identified from the GSE3467, GSE3678, and GSE33630 datasets, respectively. Volcano plots were generated for the 3 gene expression datasets for intuitive representation of the DEGs (Fig. 1a–c). The green plots represent downregulated DEGs, the red plots represent upregulated DEGs, and the black plots are not DEGs. In addition, Venn diagrams were also drawn for comparison of the number of total DEGs, upregulated DEGs, and downregulated DEGs in the three datasets. As a result, 263 common DEGs were obtained (Fig. 1d), comprising 120 coupregulated genes and 143 codownregulated genes (Fig. 1e, f).

The miRNA datasets GSE113629 and GSE73182 were also analyzed to screen deregulated miRNAs. The volcano plots showed that 16 and 2008 deregulated miRNAs were identified from the GSE73182 and GSE113629 datasets, respectively (Fig. 1g, i). Among them, 8 downregulated miRNAs and 8 upregulated miRNAs in the GSE73182 dataset, 96 downregulated miRNAs and 1912 upregulated miRNAs in the GSE113629 dataset, were identified. In addition, a Venn diagram was generated for comparison of the number of deregulated miRNAs in the two miRNA datasets; thus, 12 common deregulated miRNAs were acquired (Fig. 1h).

### Pathway enrichment analysis of common DEGs

**Fig. 2:**
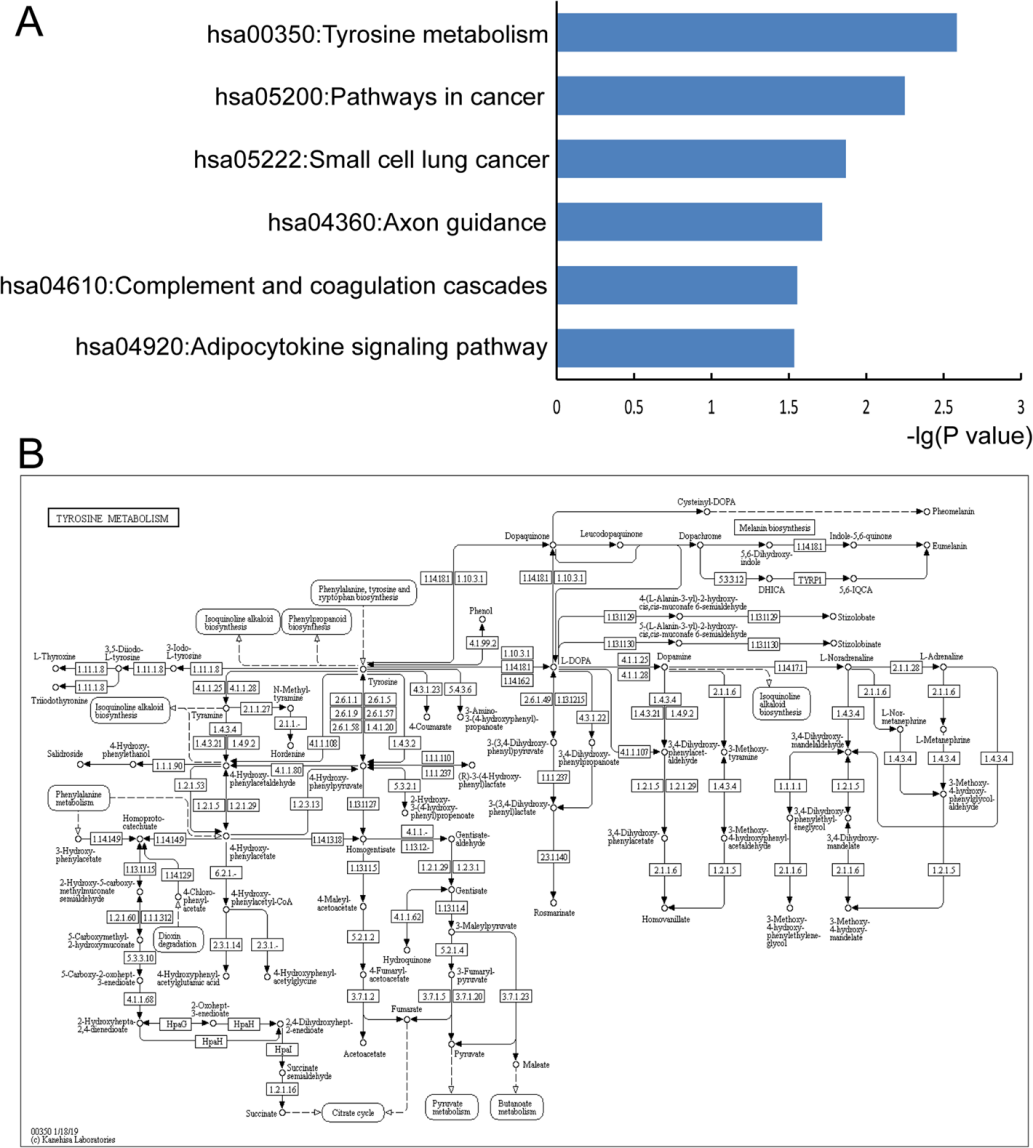
Pathway enrichment analysis of common DEGs. **a** Top six significant pathways of DEGs with KEGG enrichment (*P* < 0.05). **b** In-depth study of the top PTC-associated pathway (hsa00350: Tyrosine metabolism). DEGs, differentially expressed genes; KEGG, Kyoto encyclopedia of genes and genomes; PTC, papillary thyroid cancer

With the aid of the David database, KEGG pathway enrichment analysis was performed using common DEGs. As a result, 6 KEGG pathways (*P* < 0.05) were significantly enriched, and the top 6 pathways are shown in Fig. 2a. These common DEGs were identified to be enriched in the pathways of ‘Tyrosine metabolism’, ‘Pathways in cancer’, ‘Small cell lung cancer’, ‘Axon guidance’, ‘Complement and coagulation cascades’ and ‘Adipocytokine signaling pathway’. The most significant pathway ‘Tyrosine metabolism’ is shown in Fig. 2b. In addition, a classification of these pathways was also performed, which showed that the six pathways were divided into three major categories (Metabolism, Human Diseases and Organismal Systems) and five minor categories (Amino acid metabolism, Cancers, Development, Immune system and Endocrine system) (Table 1). These results indicated that PTC was closely related to amino acid metabolism, development, immune system, and endocrine system.

**Table 1:** Six significant KEGG pathways and their classification

Pathway	*P* value	Classification
hsa00350:Tyrosine metabolism	0.002602	Metabolism; Amino acid metabolism
hsa05200:Pathways in cancer	0.005643	Human Diseases; Cancers: Overview
hsa05222:Small cell lung cancer	0.013589	Human Diseases; Cancers: Specific types
hsa04360:Axon guidance	0.019287	Organismal Systems; Development
hsa04610:Complement and coagulation cascades	0.027889	Organismal Systems; Immune system
hsa04920:Adipocytokine signaling pathway	0.029209	Organismal Systems; Endocrine system

### GO enrichment analysis of common DEGs

**Fig. 3:**
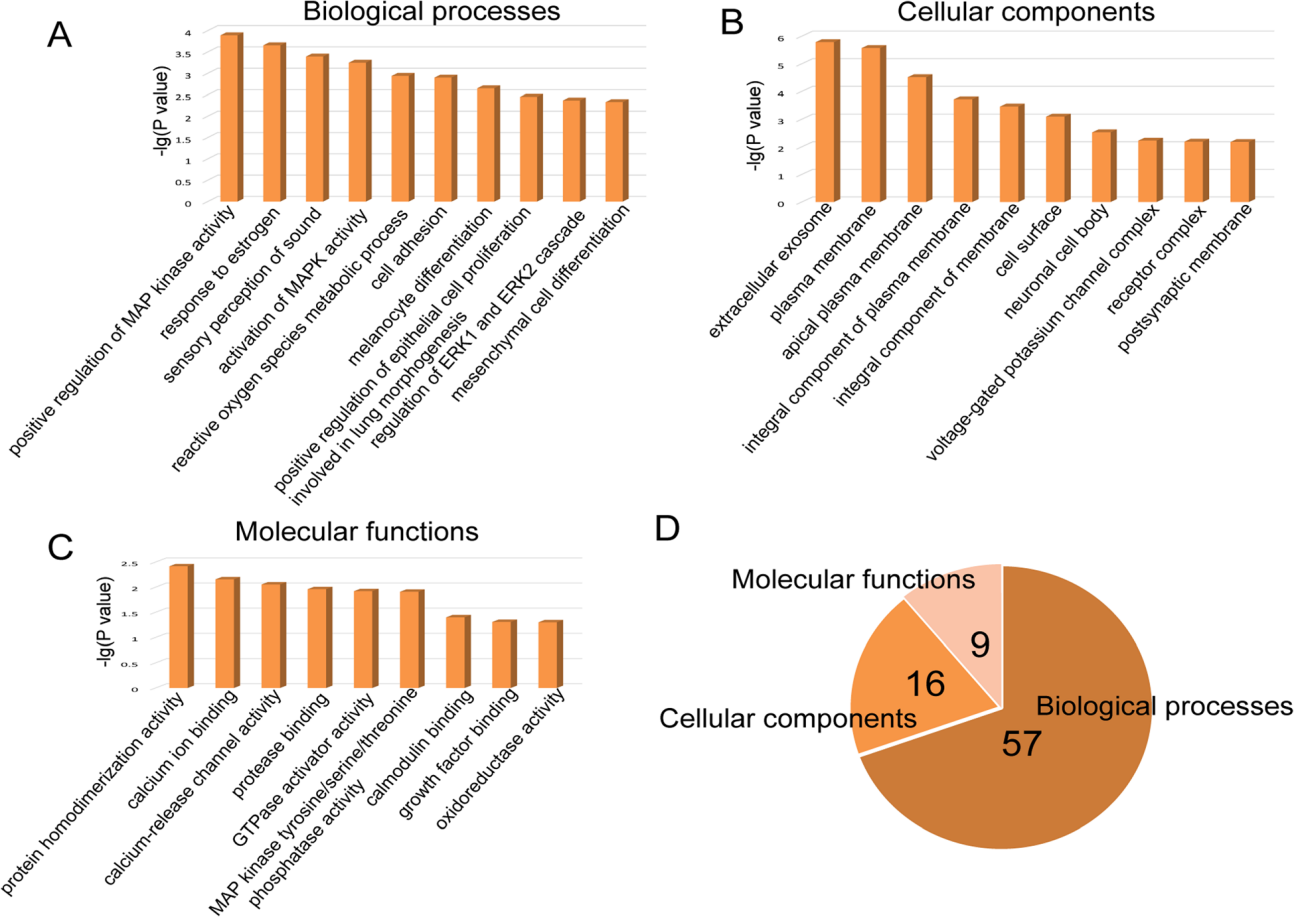
GO enrichment analysis of common DEGs. **a** The top 10 significant biological processes (*P* < 0.05). **b** The top 10 significant cellular components (*P* < 0.05). **c** The top 9 significant molecular functions (*P* < 0.05). **d** A pie chart of the proportion of the significant GO terms, including 57 biological processes, 16 cellular components and 9 molecular functions. DEGs, differentially expressed genes; GO, Gene Ontology

To better understand common DEGs, a GO enrichment analysis was also performed using DAVID. As a result, a total of 82 significant GO terms were identified with a cutoff of *P* < 0.05. The top biological processes (BPs), cellular components (CCs), and molecular functions (MFs) are shown in Fig. 3 a, b, and c, respectively. A total of 57 BPs, 16 CCs, and 9 MFs were included in the 82 significant GO terms (Fig. 3d). The top 10 BPs were ‘positive regulation of MAP kinase activity’, ‘response to estrogen’, ‘sensory perception of sound’, ‘activation of MAPK activity’, ‘reactive oxygen species metabolic process’, ‘cell adhesion’, ‘melanocyte differentiation’, ‘positive regulation of epithelial cell proliferation involved in lung morphogenesis’, ‘regulation of ERK1 and ERK2 cascade’ and ‘mesenchymal cell differentiation’. In addition, the most significant CC was the ‘extracellular exosome’, and the most significant MF was ‘protein homodimerization activity’. These results further illustrate the fundamental characteristics and functions of cell adhesion, differentiation, proliferation, and MAPK activity in PTC. Therefore, the roles of these DEGs could be better understood by analyzing these significantly enriched GO terms in PTC pathogenesis.

### Construction of the PPI network and module analysis

**Fig. 4:**
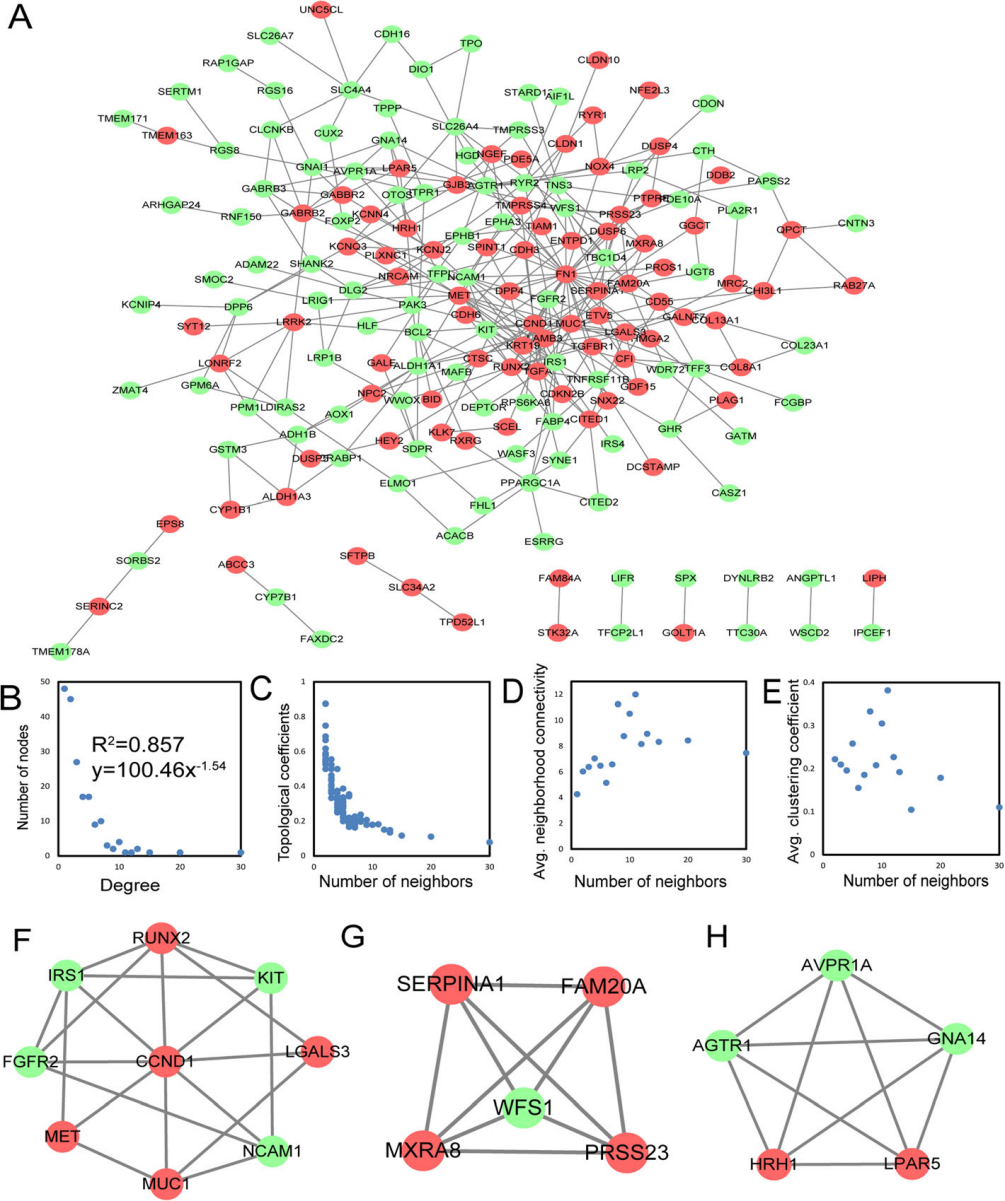
PPI network constructed from the common DEGs and their topological features and significant modules. **a** A PPI network was constructed from STRING using the 164 common DEGs. The nodes represent proteins, the edges represent the interactions of proteins and the green circles and red circles indicate downregulated and upregulated DEGs, respectively. **b**–**e** The basic topological features of the network included degrees, topological coefficients, neighborhood connectivity, and clustering coefficients. The degree distribution of this network displayed a power law distribution of f(*x*) = 100.46*x*^−1.54^ and an *R*^2^ of 0.857. **f**–**h** Three significant modules in the PPI network with MCODE scores ≥ 5. Green circles and red circles indicate downregulated and upregulated DEGs, respectively. PPI, protein-protein interaction; DEGs, differentially expressed genes; STRING, Search Tool for the Retrieval of Interacting Genes; MCODE, Molecular Complex Detection

To further explore the interaction among the 263 common DEGs, a PPI network was constructed (Fig. 4a). The PPI network contained 189 nodes and 346 edges. To explore the basic characteristics of the PPI network, the topological features of the network were analyzed in terms of degrees, topological coefficients, neighborhood connectivity, and clustering coefficients (Fig. 4b–e). It was observed that this network followed a natural rule that the majority of nodes had a low degree and that only a few nodes were highly connected with the others. Similar to other biological networks, the degree distribution of this network displayed a power law distribution of f(*x*) = 100.46*x*^−1.54^ with an *R*^2^ of 0.857, indicating that the network followed a scale-free distribution and behaved like a small-world network [28].

Furthermore, the hub genes in the PPI network with a connectivity degree > 10 were identified using the CentiScaPe v2.0 plugin for Cytoscape. The most significant 11 node degree genes were *FN1*, *CCND1*, *MET*, *RUNX2*, *IRS1*, *SERPINA1*, *KRT19*, *FGFR2*, *MUC1*, *NCAM1*, and *WFS1*. Detailed information on the 11 hub genes is summarized in Table 2. Among these 11 hub genes, *IRS1* and *WFS1* had not been previously validated in PTC, which prompted us to further investigate the role of *IRS1* and *WFS1* in PTC.

**Table 2:** Detailed information on the 11 hub genes identified in the PPI network

Gene	Full name	Overview	Expression in PTC	Reference
*FN1*	Fibronectin 1	*FN1* is involved in cell adhesion and migration processes including host defense and metastasis.	Upregulated	[29]
*CCND1*	Cyclin D1	*CCND1* has been demonstrated to interact with tumor suppressor protein Rb.	Upregulated	[30]
*MET*	MET proto-oncogene, receptor tyrosine kinase	*MET* plays a role in cellular survival, embryogenesis, and cellular migration and invasion.	Downregulated	[31]
*RUNX2*	RUNX family transcription factor 2	*RUNX2* is essential for osteoblastic differentiation and skeletal morphogenesis.	Upregulated	[32]
*IRS1*	Insulin receptor substrate 1	Mutations in *IRS1* are associated with type II diabetes and susceptibility to insulin resistance.	No relevant biological experiments	[33]
*SERPINA1*	Serpin family A member 1	The protein encoded by *SERPINA1* is an inhibitor whose targets include elastase, plasmin, thrombin, trypsin, chymotrypsin, and plasminogen activator.	Upregulated	[34]
*KRT19*	Keratin 19	*KRT19* is responsible for the structural integrity of epithelial cells. It is specifically expressed in the periderm.	Upregulated	[35]
*FGFR2*	Fibroblast growth factor receptor 2	*FGFR-2* is involved in regulating cell proliferation, migration and differentiation, as well as in the response to injury and tissue repair.	Downregulated	[36]
*MUC1*	Mucin 1, cell surface associated	Overexpression, aberrant intracellular localization, and changes in the glycosylation of *MUC1* have been associated with carcinomas.	Downregulated	[37]
*NCAM1*	Neural cell adhesion molecule 1, also known as CD56	*NCAM1* is involved in the development of the nervous system, the expansion of T cells and dendritic cells with a regulatory role in cell motility and migratory capacity of neoplastic cells.	Downregulated	[38]
*WFS1*	Wolframin ER transmembrane glycoprotein	Mutations in *WFS1* are associated with Wolfram syndrome, also called DIDMOAD (diabetes insipidus, diabetes mellitus, optic atrophy, and deafness), an autosomal recessive disorder.	No related references	[39]

The MCODE plug-in was also used to identify significant clusters. As a result, 3 clusters were identified. One cluster consisted of 9 nodes and 20 edges and included *RUNX2*, *IRS1*, *KIT*, *FGFR2*, *CCND1*, *LGALS3*, *MET*, *MUC1*, and *NCAM1*, which exhibited the highest score (Fig. 4f). Another cluster containing 9 nodes and 20 edges, including *SERPINA*, *FAM20A*, *WFS1*, *MXRA8*, and *PRSS23*, also possessed a strong connection (Fig. 4g). Furthermore, the third cluster contained 5 nodes and 10 edges and included *AVPR1A*, *AGTR1*, *GNA14*, *HRH1*, and *LPAR5* (Fig. 4h). These findings strongly suggested that the hub genes we identified could play critical roles in the pathogenesis of PTC.

### Comprehensive analysis of common deregulated miRNA and DEGs

**Fig. 5:**
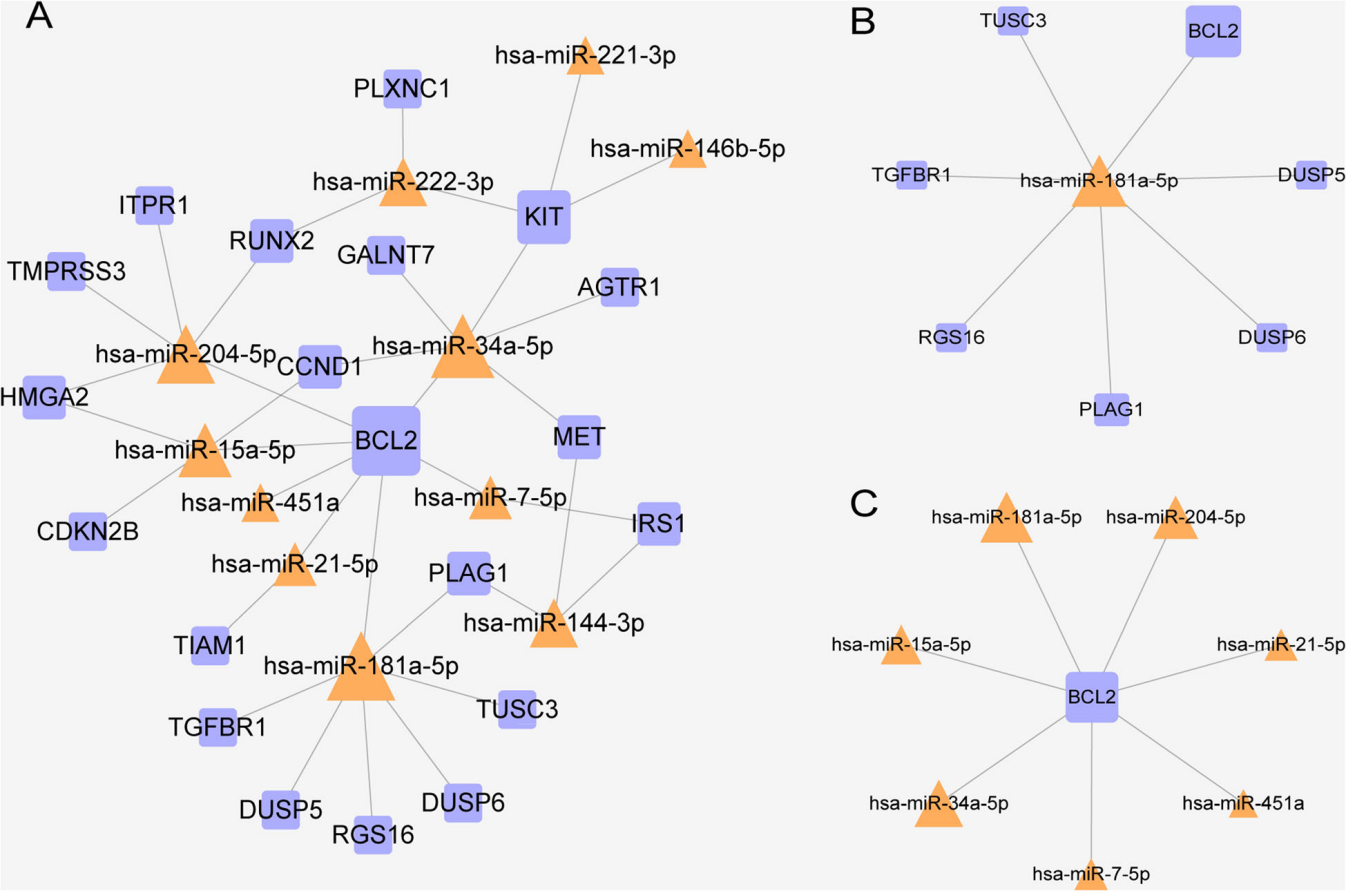
miRNA-gene regulatory network. **a** A global view of the network. The network was constructed from common DEGs and deregulated miRNAs. The network contained 11 common deregulated miRNAs, 20 common DEGs, and 35 miRNA-gene pairs. The orange triangle represents miRNA, while the blue square represents DEG. **b** A subnetwork with hsa-miR-181a-5p as the central node. **c** A subnetwork with *BCL2* as the central node. DEGs, differentially expressed genes

The miRNA-gene pairs were based on the 12 common deregulated miRNAs and 263 common DEGs. Thus, 35 miRNA-gene pairs were acquired that included 11 common deregulated miRNAs and 20 common DEGs. We constructed a miRNA-gene regulating network, and the network is shown in Fig. 5a, which illustrates that certain miRNAs play important roles in regulating DEGs. In Fig. 5a, the larger the size of the modules, the greater the degree they have. The nodes with greater degrees tend to be network hubs and are usually considered to play critical roles in maintaining the overall connectivity of the network [40]. In this network, hsa-miR-181a-5p regulated the most DEGs, while *BCL2* was targeted by the most miRNAs; in other words, hsa-miR-181a-5p and *BCL2* had the greatest degrees among miRNAs and DEGs, respectively, and they were considered to be network hubs. Therefore, we generated subnetworks with hsa-miR-181a-5p and *BCL2* as the central nodes (Fig. 5b, c). This information may be important in establishing underlying molecular mechanisms of PTC, which may be used in the development of targets for further research and diagnosis.

## Discussion

Although great progress has been made in PTC research in recent decades, the pathogenesis of PTC still needs to be further clarified. The urgent need for better treatment of PTC has sparked a search for an easy method for the early recognition of benign or malignant modules. If there is a way to find a certain deregulated gene or ncRNA for the early recognition of PTC, then the treatment of PTC could be further improved.

In our present study, PTC-associated deregulated genes and miRNAs were identified based on 3 gene and 2 miRNA expression microarray datasets. A total of 263 DEGs and 12 deregulated miRNAs were selected. Then, pathway and GO enrichment analyses were performed using the 263 DEGs to elucidate the function of PTC-associated DEGs. In addition, PPI networks were constructed to discover hub genes and core clusters. In addition, a deregulated miRNA-gene network was also constructed, and hsa-miR-181a-5p was found to regulate the most DEGs, while *BCL2* was targeted by the most miRNAs.

Pathway enrichment analysis using DEGs of PTC provided an insightful overview in elucidating the mechanism of PTC. Among the top 6 pathways, two were associated with Human Diseases; Cancers, while three were categorized into Organismal Systems and were predicted to attribute to development (axon guidance), immune system and endocrine system, and the last pathway was related to amino acid metabolism, suggesting that the pathogenesis of PTC may be closely related to amino acid metabolism, axon guidance, immune system, and endocrine system. For example, Li Y et al. discovered metabolic changes associated with PTC by nuclear magnetic resonance (NMR)–based metabolomic technique, including branched chain amino acid metabolism (leucine and valine), other amino acid metabolism (glycine and taurine) and other metabolisms of other substances, such as glycolysis, tricarboxylic acid cycle, choline metabolism, and lipid metabolism, among which amino acid metabolism function as an oncogenic substance, suggesting that amino acid metabolism may play an important part in the pathogenesis of PTC [41]. Slits, representative axon guidance molecules have been reported whose overexpression regulates the activity of Rho GTPase by inhibiting the transcriptional activity of beta-catenin and inhibiting the cell proliferation, migration, and invasion of thyroid cancer [42], which was consistent with our pathway enrichment results. In addition, a distinct tumor immune microenvironment exists in PTC, correlating with pathological aggressiveness [43]. All these findings indicated a complex mechanism of PTC involving the immune and endocrine system with the participation of amino acid metabolism and axon guidance.

To verify the interaction between the functions of DEGs identified, a PPI network was constructed in which 11 hub genes with the highest connective degree were selected that included *FN1*, *CCND1*, *MET*, *RUNX2*, *IRS1*, *SERPINA1*, *KRT19*, *FGFR2*, *MUC1*, *NCAM1*, and *WFS1*. Some of these genes have been reported to be closely associated with PTC; however, their precise roles and molecular mechanisms have not yet been fully elucidated. Fibronectin 1 (*FN1*), with the highest connective degree in this PPI network, encodes fibronectin, which is involved in cell adhesion and migration processes, including host defense and metastasis [44]. The overexpression of *FN1* is an important determinant of PTC aggressiveness [29]. In addition, *FN1* is targeted by miR-139 and functions in inhibiting tumorigenesis in PTC cells [45]. *CCND1*, short for cyclin D1, has been demonstrated to interact with tumor suppressor protein Rb [46]. Mutations, amplification, and overexpression of *CCND1*, which alters cell cycle progression, occurred frequently in a variety of tumors, including PTC, and may lead to tumorigenesis [30, 47].

**Fig. 6:**
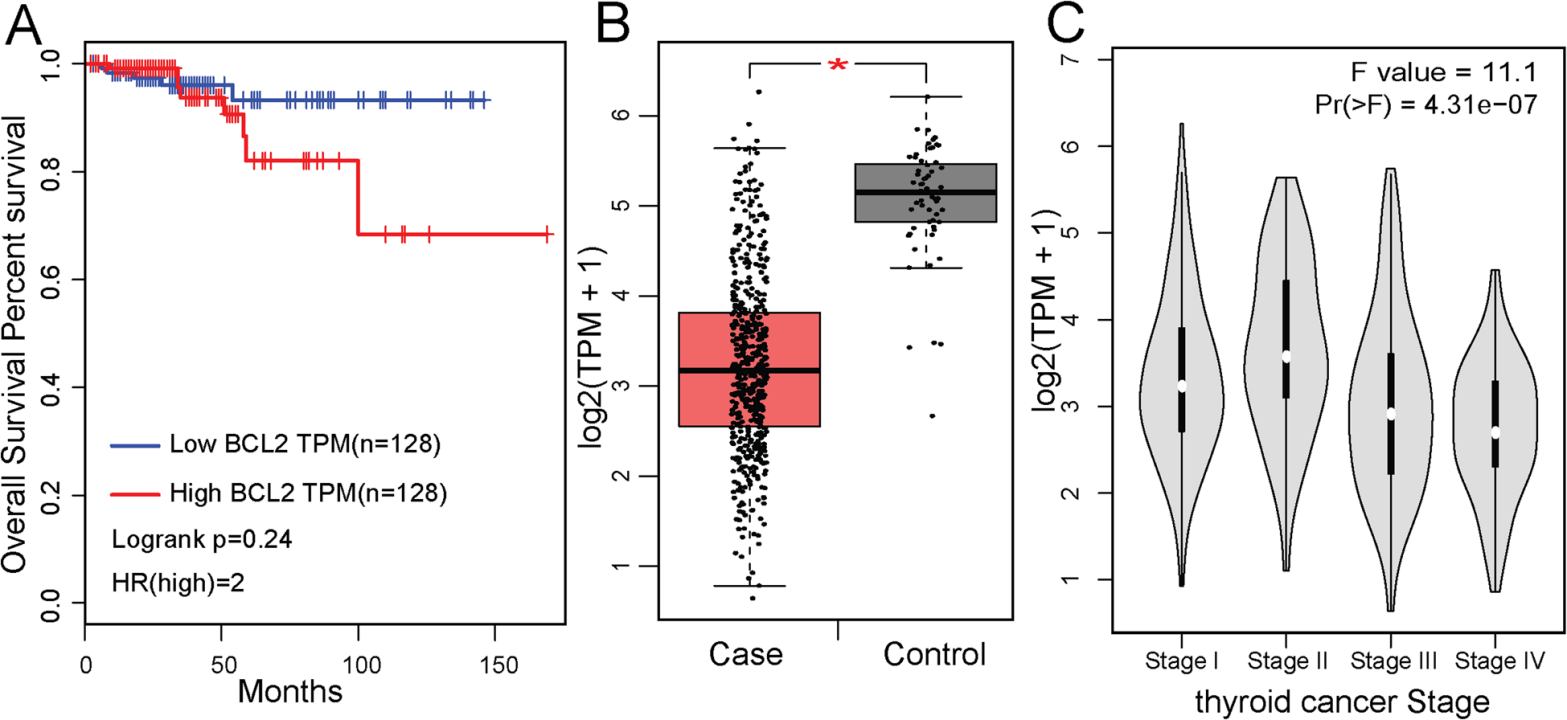
The predictive power of *BCL2* for the clinical stage and patient survival. **a** The Kaplan-Meier curve of TCGA samples categorized by the expression level of *BCL2*. **b** The boxplot of the *BCL2* expression level in the Case and Control groups. **c** Expression box diagram of *BCL2* in different tumor stages

In addition, a miRNA-gene regulatory network was constructed to further explore the association between deregulated miRNAs and DEGs in PTC. The results showed that hsa-miR-181a-5p and *BCL2* were the most impactful miRNA and gene of PTC. However, few biological studies have focused on the relationship between hsa-miR-181a-5p and PTC. By consulting the literature in PubMed, only one study was found to have reported the underlying mechanism that miR-181a-5p was oppositely expressed in the exosomes of both PTC and follicular thyroid cancer (FTC), which can help distinguish these two types of TC through comparison [48]. Similarly, studies on the relationship between *BCL2* and PTC are also very rare, and only a few studies have verified the correlation between *BCL2* and PTC. For example, the synonymous SNP rs1801018 and the G allele of the *BCL2* gene were discovered by Eun et al., and this alteration may be related to the bilaterality and multifocality of PTC [49]. It has been found that loss of *BCL2* is associated with dedifferentiation in thyroid tumors [50]; however, whether there is a connection that deregulation of *BCL2* influences PTC remains unclear. Our study provides a novel way to discover how *BCL2* and hsa-miR-181a-5p act on PTC through biological experiments. Next, we used the GEPIA database [51] to analyze the predictive power of *BCL2* for the clinical stage and survival of TCGA thyroid cancer (Fig. 6). The survival analysis results showed that there was no statistically significant association between the expression level of *BCL2* and the survival of patients with thyroid cancer. However, observed that high levels of *BCL2* expression could be correlated with a 2-fold higher mortality rate in patients than in the low-expression group (Fig. 6a, HR (high) = 2). Therefore, the expression level of *BCL2* can be used as a predictor of survival in patients with thyroid cancer. In addition, we found that in TCGA thyroid cancer data, *BCL2* was differentially expressed between cases and controls. *BCL2* was generally downregulated in tumor tissues, and this trend became more pronounced as the disease progressed, so *BCL2* also has a potential to be a dynamic signal for estimating the progression and prognosis of thyroid cancer (Fig. 6b, c).

However, there are still some limitations of this study. (i) Different methods of functional analyses have different advantages and disadvantages. In this study, we applied DAVID for functional analyses. The limitations of the DAVID tool include that it only uses the number of genes, regardless of the gene expression level or differential expression value. In addition, to obtain the genes of interest or differential expression, an artificial threshold was needed. In addition, it usually uses the most significant genes and ignores the genes with no significant differences, which may result in a loss of genes with lower significance but a more critical role, resulting in decreased detection sensitivity. (ii) Microarray data were obtained from the GEO database, not generated by the authors. (iii) This study focused on bioinformatics methods to screen candidate genes and miRNAs for PTC; however, the predicted results should be confirmed by laboratory data. Further validation with larger sample sizes and in vitro and in vivo experiments are required to confirm these results. (iv) *BCL2* has two main isoforms (1G5M and 1G5O/1GJH), and the structures of the two *BCL2* isoforms were found to be very similar [52, 53]. Further experiments could be performed to recognize the differences in the results for different isoforms of *BCL2* and may be useful for the treatment of cancers.

## Conclusions

In conclusion, we systematically identified DEGs and deregulated miRNAs through microarray datasets. Pathway and GO enrichment analysis gave us an insightful view of the functions of DEGs. Furthermore, the construction of the PPI network and miRNA-gene regulatory network provided us with hsa-miR-181a-5p and *BCL2* for further biological experiments to determine the regulatory relationship between them in PTC. As biomarkers, hsa-miR-181a-5p and *BCL2* have enormous potential for distinguishing benign or malignant nodules and guiding clinical treatments. These findings will provide important clues for investigating the pathogenesis and therapeutic methods of PTC.

## Supplementary information

**Additional file 1:** **Figure S1.** Sample analysis

## Data Availability

The datasets used and/or analyzed during the current study are available from the corresponding author on reasonable request.

## Abbreviations

BPs: Biological processes
*CCND1*: Cyclin D1
CCs: Cellular components
*CITED1*: Cbp/p300 interacting transactivator with Glu/Asp rich carboxy-terminal domain 1
DAVID: Database for Annotation, Visualization and Integrated Discovery
DEGs: Differentially expressed genes
DTCs: Differentiated thyroid cancers
FDR: False discovery rate
*FMO1*: Flavin-containing monooxygenase 1
*FN1*: Fibronectin 1
FTC: Follicular thyroid cancer
GEO: Gene Expression Omnibus
GO: Gene Ontology
*IL17RA*: Interleukin 17 receptor A
KEGG: Kyoto Encyclopedia of Genes and Genomes
*LRP4*: LDL receptor related protein 4
MCODE: Molecular Complex Detection
MFs: Molecular functions
NMR: Nuclear magnetic resonance
PPI: Protein-protein interaction
PTC: Papillary thyroid cancer
STRING: The Search Tool for the Retrieval of Interacting Genes
*TEKT4*: Tektin 4
