# Supplementary figures and images for: BCL2 and hsa-miR-181a-5p are potential biomarkers associated with papillary thyroid cancer based on bioinformatics analysis
Source: World J Surg Oncol. 2019 Dec 16;17:221. doi: 10.1186/s12957-019-1755-9 (PMC6916035; doi:10.1186/s12957-019-1755-9)

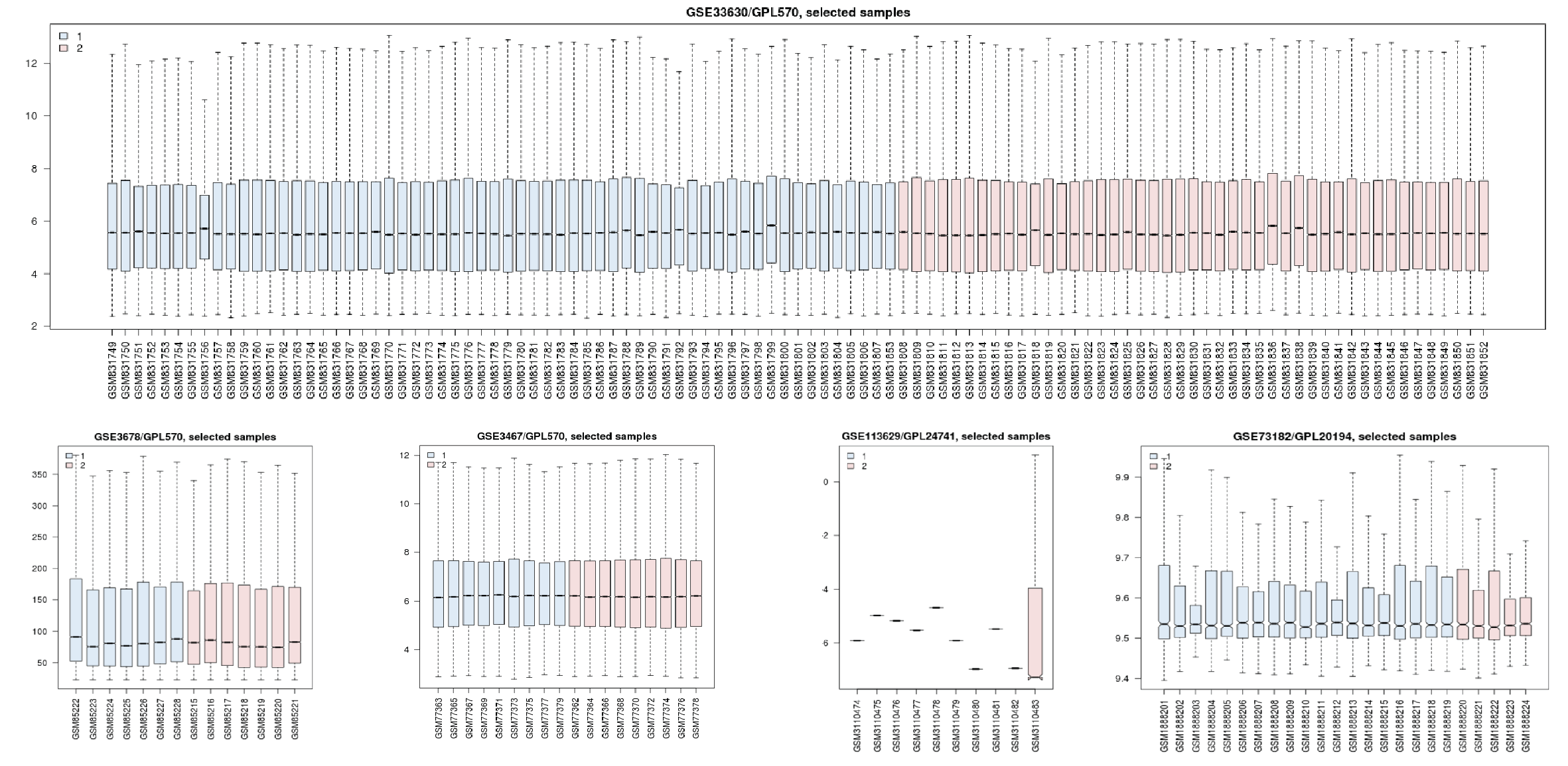

Supplement: Supplementary file 1 — Additional file 1: Figure S1. Sample analysis [file 12957_2019_1755_MOESM1_ESM.tif]
